# Genome-wide Linkage Analyses of Quantitative and Categorical Autism Subphenotypes

**DOI:** 10.1016/j.biopsych.2008.05.023

**Published:** 2008-10-01

**Authors:** Xiao-Qing Liu, Andrew D. Paterson, Peter Szatmari, The Autism Genome Project Consortium

**Affiliations:** a Program in Genetics and Genome Biology, The Hospital for Sick Children, Toronto, Ontario, Canada; b Departments of Public Health Sciences, Psychiatry and the Institute of Medical Sciences, University of Toronto, Toronto, Ontario, Canada; c Department of Psychiatry and Behavioural Neurosciences, McMaster University, Hamilton, Ontario, Canada

**Keywords:** Autism, genetic heterogeneity, IQ, language, linkage analysis, schizophrenia

## Abstract

**Background:**

The search for susceptibility genes in autism and autism spectrum disorders (ASD) has been hindered by the possible small effects of individual genes and by genetic (locus) heterogeneity. To overcome these obstacles, one method is to use autism-related subphenotypes instead of the categorical diagnosis of autism since they may be more directly related to the underlying susceptibility loci. Another strategy is to analyze subsets of families that meet certain clinical criteria to reduce genetic heterogeneity.

**Methods:**

In this study, using 976 multiplex families from the Autism Genome Project consortium, we performed genome-wide linkage analyses on two quantitative subphenotypes, the total scores of the reciprocal social interaction domain and the restricted, repetitive, and stereotyped patterns of behavior domain from the Autism Diagnostic Interview-Revised. We also selected subsets of ASD families based on four binary subphenotypes, delayed onset of first words, delayed onset of first phrases, verbal status, and IQ ≥ 70.

**Results:**

When the ASD families with IQ ≥ 70 were used, a logarithm of odds (LOD) score of 4.01 was obtained on chromosome 15q13.3-q14, which was previously linked to schizophrenia. We also obtained a LOD score of 3.40 on chromosome 11p15.4-p15.3 using the ASD families with delayed onset of first phrases. No significant evidence for linkage was obtained for the two quantitative traits.

**Conclusions:**

This study demonstrates that selection of informative subphenotypes to define a homogeneous set of ASD families could be very important in detecting the susceptibility loci in autism.

To meet the criteria for a diagnosis of autism (Mendelian Inheritance in Man [MIM] 209850), a neurodevelopmental disorder, a child must exhibit impairments in social interaction and communication, as well as show restricted and stereotyped patterns of behavior and activities (1,2). These separate criteria suggest that the diagnosis of autism is multivariate in nature. To date, many linkage studies have been performed on the discrete diagnosis of autism or autism spectrum disorders (ASD) with the aim of identifying susceptibility genes (3–11). Suggestive evidence for linkage has been found on chromosomal regions 2q, 3q, 7q, and 17q in at least two independent studies. However, very few of the above loci have reached the level suggested for genome-wide significance (12).

In comparison, autism subphenotypes may have advantages over simply employing the diagnosis of autism for genetic mapping. Subphenotypes (13) are defined as the traits that are associated with (or a part of) one of the three core autism domains and can be measured by instruments such as the Autism Diagnostic Interview-Revised (ADI-R) (14). A subphenotype may be under the control of fewer loci (and their genetic effects may be larger) and quantitative subphenotypes may be more suitable for genetic studies since autism is often conceived as a spectrum of disorders composed of several dimensions (13,15).

Family and twin studies have shown that the three core autistic domains are heritable. Based on a study of 3400 twin pairs from the general population, Ronald *et al.* (16) found high heritabilities (.78–.81) for autistic-like measurements in social-communication impairments as well as restricted, repetitive behaviors and interests using scores from the Childhood Asperger Syndrome Test. Using scores from the ADI-R and sampling affected sibships, MacLean *et al.* (17) found that the nonverbal communication domain total score was familial with an intraclass correlation coefficient (ICC) of .39 (*p* < .05); Silverman *et al.* (18) and Spiker *et al.* (19) found moderate familialities for the nonverbal communication (ICC = .19, *p* < .01; and *r* = .15, *p* < .05, respectively) and repetitive behaviors and stereotyped patterns domain total scores (ICC = .34, *p* < .001; and *r* = .22, *p* < .01, respectively); and Kolevzon *et al.* (20) found that the social interaction (ICC = .75, *p* < .0005) and communication domain total scores (ICC = .52, *p* = .01) were also familial using data from affected monozygotic twins. In addition, Sung *et al.* (21) detected moderate heritabilities for social motivation (.19) and range of interests/flexibility (.16) domains as measured by the Broader Phenotype Autism Symptom Scale from 201 nuclear families having at least two children affected with ASD. This study also found that the shared genetic variance between these two traits was low, supporting independent analyses of these two traits.

Besides measures of the three core autistic domains, other subphenotypes associated with these domains but not part of the diagnostic criteria were also found to be heritable. These included nonverbal IQ (17,19,22,23) and several aspects of language development as measured by the Vineland Communication Scale (23), verbal/nonverbal status (17–19), age of first words (24), and age of first phrases (18,24).

The heritability of these autism subphenotypes has provided a foundation for direct linkage analyses of these traits. Alarcón *et al.* (25) performed quantitative trait linkage analyses using the ADI-R age of first single words, age of first phrases, and repetitive and stereotyped behavior total score from 123 families of the Autism Genetics Resource Exchange (AGRE) and found suggestive evidence for linkage on chromosome 7q for age of first words. This evidence for linkage was supported in a follow-up study with 168 additional AGRE families (24). Evidence for linkage at the same region was also found in an independent study for age at first phrases (26). Other linkage studies on subphenotypes include a study by Chen *et al.* (27), which found a suggestive quantitative trait locus on chromosome 1 for the ADI-R nonverbal communication total score using 228 AGRE families, while Duvall *et al.* (28) performed quantitative trait linkage analysis on the Social Responsiveness Scale (29) using 100 families from AGRE and found linkage signals on chromosomes 11 and 17.

The heritability of these subphenotypes (the reduced intrafamily variance compared with the interfamily variance) also provides evidence of genetic heterogeneity in ASD. Studies have shown that some evidence for linkage for ASD was obtained only in the subsets of ASD families defined by certain subphenotypes, such as the language-related subphenotypes (30–33), developmental regression (26,34), and obsessive-compulsive behavior (35). A number of studies have also applied the ordered-subset analysis method (36) to identify subsets of families according to certain quantitative subphenotypes and found stronger linkage signals for ASD on chromosomes 7 (24), 15 (37), 19 (38), 8, and 16 (27).

Although the above genetic analyses using the autism subphenotypes have shown some promising results, the susceptibility genes for autism and the subphenotypes remain evasive, possibly due to the low statistical power of modest numbers of families. Previously, we reported linkage results for the categorical diagnosis of autism (defined in a broad and narrow way) using 1181 families from the Autism Genome Project (AGP) Consortium data (3). In the present study, we performed genome-wide linkage analyses on two quantitative subphenotypes from this sample: the reciprocal social interaction domain total score and the restricted, repetitive, and stereotyped patterns of behavior domain total score from the ADI-R. We also selected subsets of ASD families on which we performed linkage analyses using the following subphenotypes: delayed onset of first words, delayed onset of first phrases, verbal status, and IQ ≥ 70. The selection of these subphenotypes was based on the previous reports of high familiality and heritability.

## Methods and Materials

### Study Samples

The original 1397 families were collected from the 10 AGP sites in North America and Europe. For each site, the diagnosis of autism was based on the ADI-R and the Autism Diagnostic Observation Schedule or best clinical estimate (for details of the AGP data, see 3). Even though our linkage analyses used autism subphenotypes rather than the categorical diagnosis, to reduce genetic (locus) heterogeneity, families were included in the linkage analysis if they had at least two individuals diagnosed with ASD. Based on the results of Risi *et al.* (39), subjects were regarded as having ASD if they were 1) at/above the ADI-R autism cutoff on the social, communication, and repetitive behavior domains; 2) one point below the ADI-R autism cutoff on both the social and communication domains; 3) at/above the autism cutoff on the social domain but one or two points below the cutoff on the communication domain; or 4) at/above the autism cutoff on the communication domain but one or two points below the cutoff on the social domain. Details of the inclusion and exclusion criteria are provided in Supplement 1.

### Autism Subphenotypes and Covariates

The following six subphenotypes were used for the linkage analyses: a best estimate IQ and five measurements from the ADI-R, including reciprocal social interaction domain total scores (SOC); restricted, repetitive, and stereotyped patterns of behavior domain total scores (BEH); age of first words; age of first phrases; and verbal/nonverbal status. These subphenotypes were only available for the individuals with ASD.

The ADI ages of first words and phrases were specified in months for most individuals, but some individuals had been coded into groups. For example, code 993 was used for an individual who had some words then lost them. Due to the difficulties of imputing these “99x” codes into exact ages, these two variables were categorized into binary traits. Individuals who had delayed onset of first words (>24 months or the ADI-R code = 994 or 997) or first phrases (>36 months or the ADI-R code = 994 or 997) were coded as affected for the traits DelayedWord and DelayedPhrase, respectively. Table 1 in Supplement 1 lists the recoding of the 99x codes. Verbal/nonverbal status had three categories in the ADI-R—verbal, nonverbal with ≥5 words, and nonverbal with <5 words. The verbal category was treated as affected for the trait Verbal. Since different AGP sites used different instruments to measure IQ (though typically affected individuals from the same family would be tested with the same instrument), AGP required that each site provide the best estimate IQ in three categories, <50, 50 to 69, and ≥70. The individuals with ASD IQ ≥ 70 were treated as affected for the trait IQ ≥ 70.

Associations between the pairs of the autism subphenotypes were tested. Chi-square tests were used for the pairs of categorical variables; nonparametric Kruskal-Wallis tests were used for the pairs of categorical and continuous variables, while Spearman rank correlation tests were used for the pairs of continuous variables. The effects of the six potential covariates on SOC and BEH were tested using mixed linear models with family as a random effect (SAS v 9.1, SAS Institute, Cary, North Carolina). There were four categorical covariates: AGP site, gender, best estimate IQ (in three categories, <50, 50–69, and ≥70), and verbal/nonverbal status (in three categories, verbal, nonverbal with ≥5 words, and nonverbal with <5 words). There were two continuous covariates: the age and calendar year of ADI-R completion. These covariates were chosen based on the literature and our preliminary review of the data. Due to nonnormality, rank transformation was applied to SOC and Box-Cox transformation to age of ADI-R completion.

### Linkage Analysis

The genotypes were obtained using the Affymetrix (Santa Clara, California) 10K single nucleotide polymorphism (SNP) arrays at the Translational Genomics Research Institute (3). Detailed genotyping methods are available at http://www.affymetrix.com/products/arrays/specific/10k.affx, and details of quality control can be found in Supplement 1. A total of 5371 tag SNPs were selected for linkage analyses so that they were not in strong linkage disequilibrium with each other (maximum D' = .6 with a mean distance of .68 cM and SD of 1 cM and a mean minor allele frequency of .31 with SD = .12) (40). The Rutgers genetic map (http://compgen.rutgers.edu/maps/) (41) was used as the basis for linear interpolation for the locations of the Affymetrix 10K SNPs with the physical locations from National Center for Biotechnology Information (NCBI) Build 35 (42). Because Merlin (http://www.sph.umich.edu/csg/abecasis/Merlin/index.html) assumes a no-interference model, the Kosambi map was converted into the Haldane map for linkage analyses while all results were reported on the Kosambi scale. The marker allele frequencies were calculated using the founders from the inferred Caucasian families by Haploview (http://www.broad.mit.edu/mpg/haploview/index.php) (43).

Variance component linkage analyses were applied to the quantitative subphenotypes SOC and BEH using Merlin (v1.0.1) (44). Multipoint nonparametric linkage analyses (NPL) from Merlin were applied to the ASD families that had two or more affected individuals defined by the four binary subphenotypes—DelayedWord, DelayedPhrase, Verbal, and IQ ≥ 70 (45). The NPL results were presented as logarithm of odds (LOD) scores under the exponential allele-sharing model (46). Since all the affected individuals defined by the four binary subphenotypes had ASD, the linkage analyses were for both the subphenotypes and ASD using a subset of families that met the criteria. To compare the linkage results of the subsets with the results of the whole data, a linkage analysis on the diagnosis of ASD from the whole data was also performed. LOD scores as well as asymptotic *p* values were reported for these linkage analyses. For the most significant linkage results from the subset analyses, the FLexible Ordered SubSet (FLOSS v1.4.1, http://www.stat.auckland.ac.nz/∼browning/floss/floss.htm#osa) software was used to generate empirical *p* values (47). More details are provided in the Supplement 1.

## Results

### Study Families

There were 976 families selected for final analyses (details can be found in the Supplement 1). The descriptive statistics for the individuals from all the ASD families and the subsets of ASD families are provided in Table 1. This sample was most similar to the “ASD all” family sample in our previous report (3).

### Autism Subphenotypes and Covariates

Table 2 lists the associations between the subphenotypes used in this study. The subphenotypes SOC and BEH were significantly correlated (*r* = .28, *p* < .0001). The associations between the two quantitative traits and the binary traits were different. The subphenotype SOC was positively associated with DelayedWord and DelayedPhrase but negatively associated with Verbal and IQ ≥ 70 (all *p* < .0001). In other words, the mean scores for SOC were higher in the affected groups defined by DelayedWord and DelayedPhrase than the mean scores in the unaffected group but were lower in the affected groups defined by Verbal and IQ ≥ 70 than the mean scores in the unaffected group. On the other hand, BEH was not associated with DelayedWord (*p* = .7), DelayedPhrase (*p* = .6), and IQ ≥ 70 (*p* = .9) but was positively associated with Verbal (the verbal group had higher BEH scores than the scores in the nonverbal group with *p* < .0001). As expected, DelayedWord and DelayedPhrase were positively associated with each other (if an individual had delayed onset of first words, he/she would be more likely to have delayed onset of first phrases) and Verbal and IQ ≥ 70 were positively associated with each other, but the two sets of traits were negatively associated with each other (all *p* < .0001).

Four of the six potential covariates were significantly associated with SOC (*p* < .05) using a mixed linear model. These covariates were AGP site, verbal/nonverbal status, age of ADI-R completion, and best estimate IQ and together they accounted for 23% of the total variance in the model. The covariates AGP site, verbal/nonverbal status, age of ADI-R completion, and gender were significantly associated with BEH (*p* < .05) and together accounted for 12% of its total variance. The final set of covariates was selected based on both the strength of association with the subphenotypes and the number of missing values for a particular covariate. For example, best estimate IQ was a very important covariate for SOC in the simple mixed linear model. When covariates verbal/nonverbal status, AGP site, and age of ADI-R completion were included in the multiple model, the association between best estimate IQ and SOC was still significant but with IQ accounting for less than 1% of the total variance of SOC. Since the inclusion of best estimate IQ would reduce the sample size by approximately one quarter due to missing values in IQ, it was not included as a covariate for SOC. Tables 2 and 3 in Supplement 1 list the effects of the selected covariates for SOC and BEH, respectively. The heritability estimate was .35 (*p* = 5 × 10^−9^) for SOC and .52 (*p* = 5 × 10^−17^) for BEH.

### Linkage Analysis

**Figure 1:**
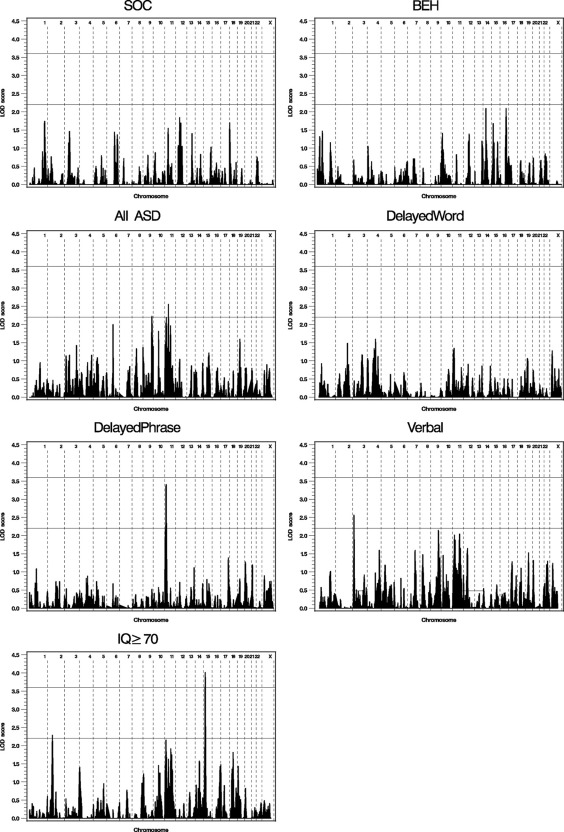
Genome-wide linkage analysis results for the seven subphenotypes. The vertical reference lines separate the chromosomes. The horizontal reference lines are at LOD score = 2.2 as the suggestive linkage threshold and LOD score = 3.6 as the significant linkage threshold. LOD, logarithm of odds; other abbreviations as in Table 1.

**Figure 2:**
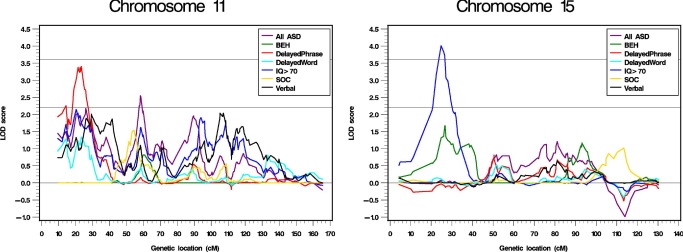
Highlighted linkage analysis results for chromosomes 11 and 15. The horizontal reference lines are at LOD score = 2.2 as the suggestive linkage threshold and LOD score = 3.6 as the significant linkage threshold. ASD, autism spectrum disorders; BEH, behavior domain total scores; LOD, logarithm of odds; SOC, social interaction domain total scores.

The genome-wide linkage results for the seven traits are illustrated in Figure 1 with the strongest linkage signals for each trait listed in Table 3. For quantitative traits SOC and BEH, no chromosomal region reached a LOD score of 2.2, the genome-wide suggestive linkage threshold. For the subset analyses, the most significant linkage signal was on chromosome 15q13.3-q14 for IQ ≥ 70 (LOD score = 4.01, *p* = .00001, δ [the locus-specific effect size] = .25) (Figure 2). Interestingly, there was also weak evidence for linkage for BEH at the same region (LOD score = 1.67, *p* = .003). The next strongest linkage signal was on chromosome 11p15.4-p15.3 for DelayedPhrase (LOD score = 3.40, *p* = .00004, δ = .19) (Figure 2). In this region, the LOD scores were 1.34 (*p* = .007) for DelayedWord, 1.89 (*p* = .002) for Verbal, and 2.15 (*p* = .0008) for IQ ≥ 70. There was also a LOD score of 2.18 (*p* = .0008) at this region when all the ASD families were used. The linkage results for individual chromosomes are provided in Figure 1 in Supplement 1.

Using permutation tests, for the most significant linkage result on chromosome 15 (LOD score = 4.01, *p* = .00001 for the ASD families with IQ ≥ 70 and LOD score = .08, *p* = .3 for all the ASD families), the probability that a subset of 313 families randomly selected from all the ASD families could reach a LOD score of 4.01 at this locus was .0006 with a 95% confidence interval of .0004 to .001. For the most significant linkage result on chromosome 11 (LOD score = 3.40, *p* = .00004 for the ASD families with DelayedPhrase and LOD score = 1.75, *p* = .002 for all the ASD families), the probability that a subset of 412 families randomly selected from all the ASD families could reach a LOD score of 3.40 at this locus was .03 with a 95% confidence interval of .02 to .05.

## Discussion

The two most significant chromosomal regions (11p15.4-p15.3 with a 1-LOD interval of 19–26 cM and 15q13.3-q14 with a 1-LOD interval of 22–28 cM) in this study were also identified in several previous studies even though their signals were not as strong. Spence *et al.* (33) showed evidence for linkage to the same regions on chromosome 11 (20–55 cM with NPL scores > 2.0) using all ASD families and on chromosome 15 (20–25 cM with NPL scores > 1.5) using language-delayed families. Evidence for linkage was also reported for the chromosome 11 region (20–30 cM with *Z* scores > 3.0) in a study by Duvall *et al.* (28) using a quantitative trait from the Social Responsiveness Scale. Both of the above studies (28,33) used families from AGRE. In the present study, 132 (32%) of the ASD families with DelayedPhrase and 67 (21%) of the ASD families with IQ ≥ 70 were from AGRE. The exact number of overlapping families between our AGRE data and those in the two previous studies is unknown. To test if our linkage results were independent confirmation of the previous reported linkage signals, we repeated the linkage analysis without any of the AGRE families. The linkage signals remained at both loci with the LOD scores changed from 3.40 to 2.78 (*p* = .0002) for the locus on chromosome 11 using the ASD families with DelayedPhrase and from 4.01 to 2.59 (*p* = .0003) for the locus on chromosome 15 using the ASD families with IQ ≥ 70.

Our linkage signal at the 15q13.3-q14 region (1-LOD interval 22–28 cM) is about 10 cM (or 6–7 Mb) telomeric to the 15q11-q13 region, which has been a focus of many association studies due to the interests in the γ-aminobutyric acid receptor α, β, and γ subunit genes in this region (48–50). Most interestingly, markers at the 15q13.3-q14 region have also been linked with an endophenotype of schizophrenia, P50 sensory gating disorder (51), and with schizophrenia itself (52,53). The most studied candidate gene in this region is α7-nicotinic cholinergic receptor (CHRNA7), which controls the excitability of local neuronal circuitries in the human cerebral cortex (54). In this study, the flanking SNPs for CHRNA7 (at 22.6–22.9 cM) were rs953325 at 22.4 cM (LOD score = 3.17, *p* = .00007) and the most significant rs1454985 at 24.8 cM (LOD score = 4.01, *p* = .00001).

Autism spectrum disorders and schizophrenia are two distinct diseases according to DSM-IV (1) and ICD-10 (2). However, studies have shown that these two disorders share a number of phenotypic features, including impairments in social cognition (55) and theory of mind (56,57). There is also evidence that adult ASDs who are relatively high functioning and verbal are more likely to present schizophrenic features, especially of the disorganized subtype (58). In addition, recent studies have reported common genes that are involved in both ASD and schizophrenia, for example, association of gene DISC1 (disrupted in schizophrenia 1) (59) and copy number variations in gene NRXN1 (neurexin 1) (60). The overlap of the clinical features and genes indicate that shared common pathogenic mechanisms may contribute to the liability for both ASD and schizophrenia. If our finding at this locus is replicated, further study will be needed to determine if the linkage evidence in this region for high-functioning ASD and for schizophrenia reflects the same biological pathways for some common intermediate phenotype(s) between these two diseases or if there is a different locus underlying each disorder within this region.

Caution should be taken when interpreting the results of this study. First, the linkage signals on chromosomes 11 and 15 may be due to either a subset of ASD cases with delayed onset of first phrases and normal or high IQ or more general language and IQ loci, which also exist in the absence of ASD. Second, the results are based on a total of seven genome-wide scans for seven traits. Because of the correlations between the traits and the subset analyses, the final number of tests is equivalent to about 5.75 independent genome-wide scans (61). Strictly speaking, none of the LOD scores reached the significant threshold needed for 5.75 independent genome scans. Third, even though we have provided both the locus-specific effect sizes and the linkage locations for the two most significant linkage signals on chromosomes 11 and 15, these estimates should be interpreted with caution because it has been shown that for genome-wide studies, regardless of the nature of phenotypes and the analytic methods, the estimates of locus-specific effect size tend to be inflated (62). In addition, according to the study by Cordell (63), the variance for a linkage location could be very large for a study with moderate sample size (e.g., 313 ASD families with IQ ≥ 70) and moderate locus-specific sibling relative risk.

Using the quantitative subphenotypes, SOC and BEH, we did not find significant linkage signals, even though we found reasonably high heritabilities for both traits (.35 for SOC and .52 for BEH). Ascertainment bias, where a pedigree was selected only if there were at least two individuals above thresholds for both the social and communication total scores, may have a large impact on heritability estimates (64). No ascertainment correction was performed since there is no efficient method for the complex ascertainment used in this study and no normative data from a general population sample exist for these two quantitative subphenotypes. In addition there may simply not be enough variation among the ASD patients to detect linkage. This is especially the case for SOC, where affected subjects had to reach a threshold to be included. In contrast, no such threshold exists for BEH. It is also important to remember that these domain total scores are summaries of the scores from many items that themselves may be heterogeneous and belong to different dimensions, so the quantitative traits we used may be as complex as the binary diagnosis of autism (65).

In addition, in a multisite study of this nature, variation in ascertainment by site may add further complexity to the analysis. To identify loci that specifically affect SOC and BEH, the effects of age of ADI-R completion, gender, verbal/nonverbal status, and AGP site on the phenotypes were removed in the linkage analyses (Tables 2 and 3 in Supplement 1). However, one could argue that the effects of AGP site, identified here as a covariate, should not be removed. In a multisite genetic study, differences in a phenotype across sites could be caused by measurement error and/or by true differences in the severity of phenotypes among affected subjects. We suspect that the differences in SOC and BEH across the AGP sites might be caused by the latter, since all sites have demonstrated good reliability; of note, individual AGP sites have also recruited families from different clinical centers. Due to these arguments, we performed linkage analyses without the AGP site as a covariate (but with the adjustments for gender, verbal/nonverbal status, and age of ADI-R completion). Without the AGP site as a covariate, the heritability for SOC changed from .35 to .50 and from .52 to .62 for BEH. However, the linkage results did not change dramatically with the ranges of the differences between –.4 to .4 for SOC and between –.4 to .5 for BEH and the highest LOD scores changed from 1.84 to 1.68 for SOC and from 2.09 to 2.06 for BEH.

Even though the analysis method using subsets of ASD families may be limited by chance findings due to reshuffling of the families and by decreased power due to reduction of sample size, the two most significant linkage signals on chromosomes 11 and 15 (both were reported in the previous studies of autism [28,33] and one [on chromosome 15] was linked to schizophrenia [52,53]) show that it is still a potentially useful method to overcome genetic heterogeneity. Autism spectrum disorder families with IQ ≥ 70 may represent a more genetically homogenous group, while the families that have rare single gene disorders, undetected chromosomal abnormalities, or de novo copy number variations tend as a whole to have lower IQ (66) and including them in a linkage analysis may reduce power.

This study is our first attempt at using the pooled multisite data to localize genetic locations using autism subphenotypes. It is apparent that pooling data from multiple sources to increase sample size is not a panacea due to the possible presence of genetic heterogeneity (67,68). However, this pooled sample did provide a larger base for us to select phenotypically homogenous subgroups, especially the ASD families with IQ ≥ 70. Future genetic studies may be improved by using the ADI-R item scores or factors derived from the item scores rather than the domain total scores due to the drawbacks discussed above. In addition, a quantitative IQ measurement may be a better heterogeneity-informative subphenotype than the categorical IQ used in this study.

### Consortia Members

*The Autism Genetics Cooperative: Canagen: SEB, JB, X-QL, ADP, WR, PS, SWS, APT, and LZ; AGC Data Coordinating Center (Battelle Center for Mathematical Medicine, The Research Institute at Nationwide Children's Hospital): CBa and VJV; Miami Institute for Human Genomics: MLC, JRG, and MAP-V; Paris Autism Research International Sibpair (PARIS) Study: CBe, CG, and MLe; Seaver Autism Research Center: JDB and KLD; Stanford University: JH and LL; Vanderbilt University: JLH and JSS; University of North Carolina/University of Iowa: JP, VS, and THW.*

*The Autism Genetic Resource Exchange Consortium: RMC, DHG, and JM.*

*The Collaborative Programs of Excellence in Autism (CPEA) and Autism Centers of Excellence (ACE): HC, BD, GD, AE, OK, JM, GDS, EMW, and WMM.*

*The International Molecular Genetic Study of Autism Consortium (IMGSAC): France: CM, BR, and KW; Germany: SB, CMF, SMK, AP, and FP; Greece: KP and JT; Italy: AB and EM; The Netherlands: HVE and MdeJ; UK: AJB, GB, PFB, TB, ALeC, JG, JAL, MLa, APM, HM, AP, JRP, KR, MLR, EW, and SW; USA: CC, EHC, SJG, VH, BLL, CL, JS, and FV; Canada: EF.*

*Other AGP sites: Dublin: SE, AG, LG, and MG; Indiana (USA): CJM, JIN, and DP; Portugal: GO and AMV.*

*Drs. Xiao-Qing Liu, Andrew D. Paterson, Peter Szatmari, and all participating members of AGP reported no biomedical financial interests or potential conflicts of interests.*

## Figures and Tables

**Table 1:** Descriptive Statistics for the Individuals from All the ASD Families and from the Subsets of ASD Families

	All ASD	DelayedPhrase	DelayedWord	Verbal	IQ ≥ 70
Gender					
Male	1618 (80)	668 (79)	470 (79)	768 (81)	523 (81)
Female	407 (20)	173 (21)	128 (21)	179 (19)	122 (19)
Verbal/Nonverbal Status					
Verbal	1337 (66)	479 (57)	339 (57)	947 (100)	565 (88)
Non-verbal	685 (34)	360 (43)	259 (43)	0 (0)	80 (12)
Best Estimate IQ					
≥70	932 (67)	322 (58)	240 (63)	593 (83)	645 (100)
<70	463 (33)	235 (42)	141 (37)	118 (17)	0 (0)
Onset of First Words					
≤24 months	912 (47)	215 (26)	0 (0)	521 (56)	339 (53)
>24 months	1032 (53)	604 (74)	598 (100)	414 (44)	299 (47)
Onset of First Phrases					
≤36 months	647 (34)	0 (0)	47 (8)	428 (46)	282 (45)
>36 months	1256 (66)	841 (100)	518 (92)	504 (54)	348 (55)
AGP Site					
AGRE	572 (28.2)	272 (32.3)	212 (35.5)	234 (24.7)	141 (21.9)
VANDERBILT	93 (4.6)	39 (4.6)	23 (3.9)	48 (5.1)	0 (0)
IMGSAC	445 (22.0)	212 (25.2)	138 (23.1)	220 (23.2)	223 (34.6)
DUKE	105 (5.2)	32 (3.8)	21 (3.5)	55 (5.8)	20 (3.1)
CANAGEN	186 (9.2)	60 (7.1)	54 (9.0)	67 (7.1)	78 (12.1)
INSERM	73 (3.6)	36 (4.3)	24 (4.0)	30 (3.2)	22 (3.4)
STANFORD	163 (8.1)	36 (4.3)	35 (5.9)	73 (7.7)	22 (3.4)
CPEA	276 (13.6)	94 (11.2)	57 (9.5)	173 (18.3)	110 (17.0)
UNC	93 (4.6)	50 (6.0)	32 (5.3)	45 (4.7)	29 (4.5)
MT. SINAI	19 (0.9)	10 (1.2)	2 (0.3)	2 (0.2)	0 (0)
Age of ADI-R Completion (month)	101 ± 67	103 ± 69	97 ± 68	116 ± 67	104 ± 50
SOC	22.0 ± 5.6	23.1 ± 5.0	22.9 ± 5.2	21.4 ± 5.7	21.3 ± 5.7
BEH	6.2 ± 2.5	6.1 ± 2.5	6.0 ± 2.5	6.7 ± 2.7	6.3 ± 2.7

Values are count (percentage) or mean ± standard deviation. ADI-R, Autism Diagnostic Interview-Revised; AGP, Autism Genome Project; AGRE, Autism Genetics Resource Exchange; ASD, autism spectrum disorders; BEH, behavior domain total scores; CANAGEN, Canadian Autism Genetics; CPEA, Collaborative Programs of Excellence in Autism; DelayedPhrase, delayed onset of first phrases; DelayedWord, delayed onset of first words; IMGSAC, International Molecular Genetic Study of Autism Consortium; INSERM, Institute National de la Santé et de la Recherche Médicale; IQ ≥ 70, best estimate IQ ≥ 70; SOC, social interaction domain total scores; UNC, University of North Carolina; Verbal, verbal category.

**Table 2:** Associations Between the Autism Subphenotypes

	SOC	BEH	DelayedPhrase	DelayedWord	Verbal	IQ ≥ 70
SOC	—	2025	1905	1946	2024	1395
BEH	.28 (< .0001)	—	1903	1944	2022	1393
DelayedPhrase	184.9 (< .0001)	.3 (.6)	—	1870	1903	1338
DelayedWord	88.7 (< .0001)	.1 (.7)	582.0 (< .0001)	—	1944	1360
Verbal	133.7 (< .0001)^a^	68.9 (< .0001)	198.5 (< .0001)^a^	94.6 (< .0001)^a^	—	1393
IQ ≥ 70	83.5 (< .0001)^a^	.03 (.9)	90.1 (< .0001)^a^	29.9 (< .0001)^a^	364.1 (< .0001)	—

The sample sizes are in the upper triangle and the strength of associations and *p* values are in the lower triangle. The strength of associations is calculated by correlation coefficient between SOC and BEH; chi-square statistic among DelayedPhrase, DelayedWord, Verbal, and IQ ≥ 70; and Kruskal-Wallis chi-square statistic between the quantitative traits (SOC and BEH) and the categorical traits (DelayedPhrase, DelayedWord, Verbal, and IQ ≥ 70). BEH, behavior domain total scores; DelayedPhrase, delayed onset of first phrases; DelayedWord, delayed onset of first words; IQ ≥ 70, best estimate IQ ≥ 70; SOC, social interaction domain total scores; Verbal, verbal category status.

a Negative association.

**Table 3:** The Most Significant Linkage Signal(s) for Each Trait

	Number of Families	Chromosome Location	SNP^a^	LOD (*p*)
SOC	976	12q13.11	rs727363	1.84 (.002)
BEH	975	14q22.1	rs1986410	2.09 (.001)
All ASD	975	9q34	rs1544105	2.22 (.0007)
		11p13	rs2421826	2.55 (.0003)
DelayedPhrase	412	11p15.4-p15.3	rs1394119	3.40 (.00004)
DelayedWord	290	4q27	rs1513721	1.59 (.003)
Verbal	461	3p26.1	rs2167037	2.56 (.0003)
IQ ≥ 70	313	2p16.3	rs1996970	2.28 (.0006)
		15q13.3-q14	rs1454985	4.01 (.00001)

More results are shown for a trait if there are more loci that have LOD scores > 2.2 (the suggestive linkage threshold for one genome-wide linkage analysis). ASD, autism spectrum disorders; BEH, behavior domain total scores; DelayedPhrase, delayed onset of first phrases; DelayedWord, delayed onset of first words; IQ ≥ 70, best estimate IQ ≥ 70; LOD, logarithm of odds; SOC, social interaction domain total scores; SNP, single nucleotide polymorphism; Verbal, verbal category status.

a SNP is the one at the position with the maximum LOD score.
