# Supplementary material for: Genome-wide Linkage Analyses of Quantitative and Categorical Autism Subphenotypes
Source: Biol Psychiatry. 2008 Oct 1;64(7):561–70. doi: 10.1016/j.biopsych.2008.05.023 (PMC2670970; doi:10.1016/j.biopsych.2008.05.023)
Supplement: Supplementary data [file mmc1.doc]

**Subjects and methods**

Study samples

We excluded individuals who were non-ASD by the clinicians’ best estimate diagnosis (n = 22), and individuals whose age of ADI completion was before 24 months (n = 1) or whose ADI scores were from the 1988 version due to different maximum total domain scores between ADI and ADI-R (n = 3). In addition to the above sample exclusion criteria, only families which were inferred to be of Caucasian origin by the computer program ‘smartpca’ in the Eigensoft package (v1.0) were included in the linkage analyses (1). The ethnicities were inferred using the founders or one non-founder per family if there was no founder genotyped in the family. The families were divided into Caucasians, non-Caucasians or mixed based on the first two principal components.

PREST (v3.02) was used to check the pairwise relationships within the pedigrees (2) and RELPAIR (v2.0.1) was used to check the pairwise relationship between the individuals from different pedigrees (3). Since both programs rely on allele frequencies to calculate the relationships and wrong allele frequencies can cause false positive results, we chose 1,104 SNPs which had small variations across the inferred ethnicity groups (within 0.1 of the allele frequencies from the Caucasian group). For PREST, pedigree errors, such as wrong relationships and related parents, were determined based on the p value (p ≤ 0.00001 in any of the PREST statistics) as well as support from other relationships within the same pedigree. For RELPAIR, a pair of individuals was considered related if the likelihood ratio between the putative and inferred relationships was ≥ 10^6^ and if there was support from other relationships between the pedigrees.

Genotype quality control

The genotypes for an individual were set to missing if there were > 20% of missing genotypes (n = 9). A SNP was excluded if it had > 10% of missing genotypes (551 out of the original 9,476 SNPs), minor allele frequency < 0.05 (791 SNPs) or Hardy-Weinberg equilibrium test p value < 0.005 (434 SNPs) calculated using the founders from the inferred Caucasian families. Since most SNP genotype errors do not lead to Mendelian inconsistencies due to lack of information in individual SNPs, Merlin (v1.0.1) was used to detect erroneous genotypes by the information of gene flow from neighboring SNPs in a pedigree (4).

Permutation tests using FLOSS

The ‘cov’ program from FLOSS was used to assign a covariate score (i.e. the sub-phenotypes such as IQ≥70 or DelayedPhrase) for each family by setting the statistic to be minimum, the subject filter to be all affected family members who had a covariate value and the minimum number of subjects to be two. This setting selected the families only if they had at least two ‘affected’ individuals who had a covariate value. Permutation tests were used to assess the null hypothesis of independence of family linkage scores at each locus and family covariate scores by comparing the maximum linkage score from the subsets of families with the maximum linkage scores obtained from the random subsets of families.

**Results**

Study families

Of the total 1,397 families with genotypes, there were 1,182 inferred Caucasian families, 106 non-Caucasian and 109 mixed families. About 95.5% of the individuals who self-defined as ‘non-Hispanic white’ were in the inferred Caucasian group, 3.9% of them were in the mixed group and 0.6% in the inferred non-Caucasian group. Of the 1,182 Caucasian families, there were 978 families which had at least two related individuals with ASD.

Using PREST, we detected likely incorrect relationships in five Caucasian pedigrees. In two pedigrees, the parents of the affected first cousin pairs were not full sibs. The parents’ relationship was corrected to be half sibs for one family. The other pedigree was deleted due to uncertain relationship. The relationships of the affected pairs from the other three pedigrees were also changed, one from half-sib to full-sib, one from full-sib to half-sib and one from first cousins to half sibs. One duplicate family was detected using RELPAIR and was deleted. The majority of the families had two individuals with ASD (n = 910), fifty-nine families had three affected individuals, five families had four affected individuals and two families had five affected individuals. Most of the affected pairs (n = 1038) were full-sibs but there were also eighteen half-sib pairs and one parent-offspring pair.

**References**

1. Price AL, Patterson NJ, Plenge RM, Weinblatt ME, Shadick NA, Reich D (2006): Principal components analysis corrects for stratification in genome-wide association studies. *Nat Genet* 38:904-909.

2. McPeek MS, Sun L (2000): Statistical tests for detection of misspecified relationships by use of genome-screen data. *Am J Hum Genet* 66:1076-1094.

3. Epstein MP, Duren WL, Boehnke M (2000): Improved inference of relationship for pairs of individuals. *Am J Hum Genet* 67:1219-1231.

4. Abecasis GR, Cherny SS, Cookson WO, Cardon LR (2002): Merlin--rapid analysis of dense genetic maps using sparse gene flow trees. *Nat Genet* 30:97-101.

Supplemental Table S1. Recoding of the ADI ‘99x’codes for DelayedWord and DelayedPhrase

| ADI-R code | Explanation | New code | Sample size – DelayedWord (observed/used) | Sample size – DelayedPhrase (observed/used) |
| --- | --- | --- | --- | --- |
| 993 | had some words, then lost and not yet regained | missing | 36/0 | 10/0 |
| 994 | milestone not reached | affected | 196/195^1^ | 450/403^2^ |
| 996 | unknown but apparently normal | unaffected | 47/47 | 50/50 |
| 997 | unknown but apparently delayed | affected | 51/51 | 54/54 |
| 999 | unknown or not asked | missing | 11/0 | 13/0 |
| Total |  |  | 341/293 | 577/507 |

Legend to Supplemental Table S1: ^1^One individual’s age of ADI completion is before 24 months. ^2^Forty-seven individuals’ ages of ADI completion are before 36 months.

Supplemental Table S2. Covariate effects for SOC

| Covariate | Categorical level | Sample size | Estimate | Standard error | P value |
| --- | --- | --- | --- | --- | --- |
| Age of ADI completion |  | 2024 | 1.78 | 0.12 | <.0001 |
| AGP site | CANAGEN | 186 | 0.14 | 0.081 | 0.08 |
|  | CPEA | 275 | -0.010 | 0.071 | 0.9 |
|  | DUKE | 105 | -0.46 | 0.10 | <.0001 |
|  | IMGSAC | 445 | 0.15 | 0.061 | 0.02 |
|  | INSERM | 73 | -0.46 | 0.12 | 0.0001 |
|  | MT. SINAI | 19 | 0.27 | 0.22 | 0.2 |
|  | STANFORD | 162 | 0.76 | 0.085 | <.0001 |
|  | UNC | 93 | -0.013 | 0.11 | 0.9 |
|  | Vanderbilt | 94 | -0.31 | 0.11 | 0.003 |
|  | AGRE | 572 | 0 | - | - |
| Gender | Female | 407 | 0.075 | 0.048 | 0.1 |
|  | Male | 1617 | 0 | - | - |
| Verbal/non-verbal status | Non-verbal < 5 words | 403 | 0.85 | 0.053 | <.0001 |
|  | Non-verbal ≥ 5 words | 282 | 0.74 | 0.059 | <.0001 |
|  | Verbal | 1339 | 0 | - | - |

Legend to Supplemental Table S2: SOC is the rank transformed score. For the categorical covariates, the group which has the largest sample size is used as a reference.

Supplemental Table S3. Covariate effects for BEH

| Covariate | Categorical level | Sample size | Estimate | Standard error | P value |
| --- | --- | --- | --- | --- | --- |
| Age of ADI completion |  | 2022 | 2.17 | 0.33 | <.0001 |
| AGP site | CANAGEN | 184 | 0.80 | 0.22 | 0.0004 |
|  | CPEA | 275 | 0.65 | 0.20 | 0.0008 |
|  | DUKE | 105 | 0.26 | 0.28 | 0.4 |
|  | IMGSAC | 445 | 0.45 | 0.17 | 0.007 |
|  | INSERM | 73 | -0.48 | 0.33 | 0.1 |
|  | MT. SINAI | 19 | -0.19 | 0.62 | 0.8 |
|  | STANFORD | 162 | 2.20 | 0.24 | <.0001 |
|  | UNC | 93 | 0.23 | 0.30 | 0.4 |
|  | VANDERBILT | 94 | 0.19 | 0.30 | 0.5 |
|  | AGRE | 572 | 0 | - | - |
| Gender | Female | 407 | -0.55 | 0.13 | < 0.0001 |
|  | Male | 1615 | 0 | - | - |
| Verbal/non-verbal status | Non-verbal < 5 words | 403 | -0.66 | 0.14 | < 0.0001 |
|  | Non-verbal ≥ 5 words | 282 | -0.66 | 0.16 | < 0.0001 |
|  | Verbal | 1337 | 0 | - | - |

Legend to Supplemental Table S3: For the categorical covariates, the group which has the largest sample size is used as a reference; BEH, behaviour domain total scores

Supplemental Figure S1. Linkage analysis results for individual chromosomes. The horizontal reference lines are at LOD score = 2.2 as the suggestive linkage threshold and LOD score = 3.6 as the significant linkage threshold.
